# Synthesis, Antiviral and Cytotoxic Activity of Novel Terpenyl Hybrid Molecules Prepared by Click Chemistry

**DOI:** 10.3390/molecules23061343

**Published:** 2018-06-03

**Authors:** Mariano Walter Pertino, Erina Petrera, Laura Edith Alché, Guillermo Schmeda-Hirschmann

**Affiliations:** 1 Laboratorio de Química de Productos Naturales, Instituto de Química de Recursos Naturales, Universidad de Talca, 3460000 Talca, Chile; schmeda@utalca.cl; 2 Programa de Investigación de Excelencia Interdisciplinaria en Química y Bio-orgánica de Recursos Naturales (PIEI-QUIM-BIO), Universidad de Talca, 3460000 Talca, Chile; 3 Laboratorio de Virología, Departamento de Química Biológica, Facultad de Ciencias Exactas y Naturales, Universidad de Buenos Aires, C1428BGA Buenos Aires, Argentina; epetrera@qb.fcen.uba.ar (E.P.); lalche@qb.fcen.uba.ar (L.E.A.); 4 Instituto de Química Biológica de la Facultad de Ciencias Exactas y Naturales (IQUIBICEN), CONICET—Universidad de Buenos Aires, C1428EGA Buenos Aires, Argentina

**Keywords:** hybrid molecules, terpenes, antiviral, cytotoxicity, click chemistry

## Abstract

Naturally occurring terpenes were combined by click reactions to generate sixteen hybrid molecules. The diterpene imbricatolic acid (IA) containing an azide group was used as starting compound for the synthesis of all the derivatives. The alkyne group in the terpenes cyperenoic acid, dehydroabietinol, carnosic acid γ-lactone, ferruginol, oleanolic acid and aleuritolic acid was obtained by esterification using appropriate alcohols or acids. The hybrid compounds were prepared by combining the IA azide function with the different terpene-alkynes under click chemistry conditions. The cytotoxic activity of the terpene hybrids **1**–**16** was assessed against Vero cells and tumour cell lines (HEP-2, C6 and Raw 264.7). Compounds **1**, **2**, **3** and **7** showed cytotoxic activity against the tested cell lines. The antiviral activity of the compounds was evaluated against HSV-1 KOS, Field and B2006 strain. For the pairs of hybrid compounds formed between IA-diterpene (compounds **3**–**8**, except for compound **7**), a moderate activity was observed against the three HSV-1 strains with an interesting selectivity index (SI ≥10, SI = CC_50_/CE_50_) for some compounds.

## 1. Introduction

Viruses are one of the main health problems worldwide due to the diseases they can cause and the low effectiveness of the current antiviral drugs [1]. One of the most common and highly infectious virus in humans is the herpes simplex virus type 1 (HSV-1). About 3500 million people worldwide have a prevalent infection of HSV-1 [2]. This virus is associated with a variety of diseases both mild and serious and even in some cases life-threatening [3]. Recent studies have demonstrated a direct relationship between HSV-1 infection with sporadic encephalitis [2] and Alzheimer’s disease [4]. Although there are drugs approved for the treatment of HSV, such as acyclovir, routine use of this drug generates resistance, especially in immunocompromised patients and patients with stem cell transplants [5], therefore, there is a continuing need to find new chemical entities to be used as antiviral agents.

Terpenes are secondary metabolites that occur across a wide range of plant tissue types and present several biological activities. Some of them have shown remarkable antiviral [6,7,8] and cytotoxic effects [9,10,11]. In previous works, we prepared series of semisynthetic derivatives of a large number of terpenes in the search for biologically active derivatives [12,13,14,15]. The semisynthetic strategies that we have used in the search for bioactive compounds have been varied. Among them, we described the synthesis of imbricatolic acid dimers [16] and hybrid terpene and quinone molecules [17]. 

Molecular hybridization is a strategy for the discovery of new drugs that consists in the fusion of two bioactive compounds to generate a new entity called a hybrid molecule [18]. In the last two decades molecular hybridization has gained importance as a valid tool in the search for bioactive compounds. A recently review highlights hybrid molecules as privileged scaffolds to identify potential bioactive agents [19]. In that review almost 200 hybrid molecules of different nature, with high structural variety and significant activities were presented. Adopting this approach, Belluti et al. [20] synthesized ten stilbene–coumarin hybrids and evaluated their antiproliferative activity against lung carcinoma H460, squamous cell carcinoma A431 and melanoma JR8 tumour cells. Some of the prepared hybrids showed better antiproliferative activity than the reference compound (resveratrol) with pro-apoptotic activities on H460 cells. Other compounds that showed potential anticancer activity were hybrids formed by two phenolic compounds. Sashidhara et al. [21] prepared a series of 21 coumarin–chalcone hybrids with significant antiproliferative activity for several of the compounds evaluated. Recently, a synthetic strategy has been reported to combine flavonoid dimers and trimers linked by triazole ring using click chemistry [22].

In this report, we used triazole rings as linkers due to the synthetic advantages of this reaction. The triazole ring also has high aromatic stabilization that make it stable against adverse conditions including hydrolysis. In addition, in the formation of hybrid molecules triazoles can act as a simple linker or spacer or, in some cases, as an active biological entity [23]. There are numerous reports where the click chemistry technique has been used to prepare hybrid molecules with various biological effects [24,25,26].

Terpene hybrids reported in bibliography include hybrids between terpene-benzoylphenyl urea (BPU) [27], hybrid terpene-amino acid [28,29], hybrid terpene-quinone [17,30], hybrid terpene-anti-inflammatory agents [31], hybrid terpene-NSAID [32], among others. However, to date there is little information on the possible biological effect that hybrid molecules formed by the fusion of two different terpenes may have. In this work, our aim was to synthesize hybrid molecules by click chemistry reactions between the diterpene imbricatolic acid and several terpenes, including cyperenoic acid, dehydroabietinol, carnosic acid γ-lactone, ferruginol, aleuritolic acid and oleanolic acid.

## 2. Results and Discussion

**Scheme 1:**
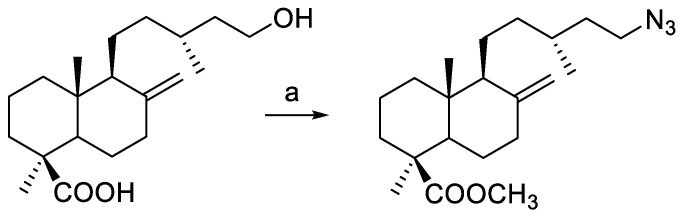
Preparation of IA-azide derivative. *Reagents and conditions*: (a) (i) CH_2_N_2_, Et_2_O, 93%; (ii) TsCl, pyridine, 76%; (iii) NaN_3_, DMF, 88%.

Naturally occurring terpenes were combined by click chemistry to obtain sixteen hybrid molecules. The diterpene imbricatolic acid (IA) with an azide group was used as starting compound for the synthesis of all the derivatives. The azide group was installed at the C-15 position of imbricatolic acid according to our previous work [16]. Briefly, IA was methylated, tosylated, and finally treated with NaN_3_ to form the azide derivative (Scheme 1). 

**Scheme 2:**
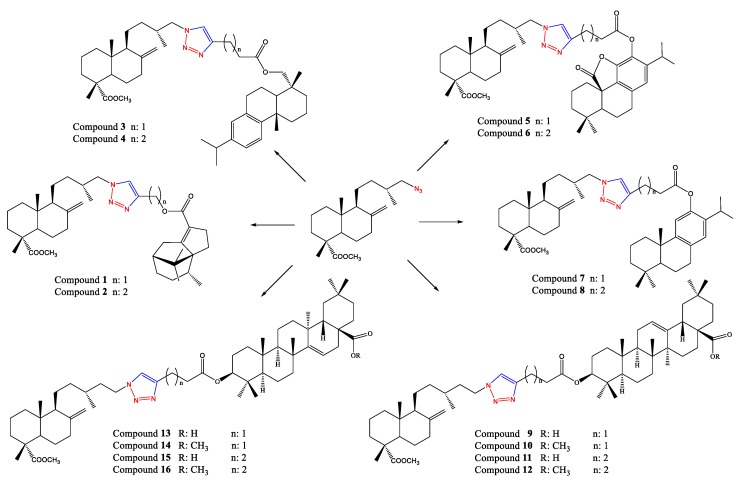
Preparation of derivatives **1**–**16**. *Reagents and conditions*: appropriate alkyne-terpene, CuSO_4_·5H_2_O, sodium ascorbate, t-BuOH/H_2_O 1:1, 53–83% yield.

The alkyne groups in the terpenes cyperenoic acid, dehydroabietinol, carnosic acid γ-lactone, ferruginol, oleanolic acid and aleuritolic acid were obtained by esterification using appropriate alcohols or acids according our previous work [13,33,34]. The alcohols used were propargyl alcohol and 3-butyn-1-ol. The acids used were 4-pentynoic acid and 5-hexynoic acid. Compounds **1**–**16** were prepared by combining the IA-azide with the different terpene-alkynes by click chemistry (Scheme 2). 

### 2.1. Cytotoxicity Assay

The cytotoxic activity of the hybrid terpenes **1**–**16** against Vero and selected tumour cell lines (HEP-2, C6 and Raw 264.7) was determined and the 50% cytotoxic concentration (CC_50_) of each compound was calculated. Only compounds **1**, **2**, **3** and **7** showed cytotoxic activity against the tested cell lines. All other compounds should be regarded as non-cytotoxic, with CC_50_ values > 1000 μM on all evaluated cell lines. The most relevant results are summarized in Table 1.

The pairs of compounds **1**–**2**, **3**–**4** and **7**–**8** differ in one CH_2_ unit in the linker. The hybrid between imbricatolic acid and cyperenoic acid (compounds **1** and **2**) showed selective cytotoxicity. Both compounds were more cytotoxic against HEP-2 cells with CC50 values of 229.7 and 48.1 μM, respectively. Compound **2** was the most cytotoxic compound among the series of derivatives against all cell lines used. Compound **1** did not show cytotoxicity against C6 and Raw 264.7 cells (see Table 1). For the pairs of compounds **3**–**4** (hybrid between IA-dehydroabietinol) and **7**–**8** (hybrid between IA-ferruginol), the activity decreased with linker length. Compound **3** presented some effect against Vero and HEP-2 cells, but not against C6 and Raw 264.7 cells. Compound **7** showed weak activity on all cell lines, with CC_50_ values ranging from 552.5 to 700 μM. Compounds **4** and **8** were not cytotoxic.

### 2.2. Antiviral Activity Screening

Most of the compounds presented moderate cytotoxicity or were inactive, which renders them suitable as potential antivirals. The antiviral activity of the compounds **1**–**16** was assessed against three different strains of HSV-1 (KOS, Field and B2006 strain). First, the 50% cytotoxic concentration (CC_50_) of the compounds was calculated using Vero cells. It is prerequisite that antiviral compounds do not cause significant damage to host cells. The results are presented in Table 2. The most cytotoxic hybrid was compound **2** (CC_50_: 216.2 μM), followed by compound **3** with CC_50_: 667.5 μM. All other hybrids were not cytotoxic.

The results showed similar antiviral activity against the HSV-1 KOS strain, with EC_50_ values of the hit compounds between 96.2–118.4 μM and with selectivity indexes (SI = CC_50_/CE_50_) >9 for compounds **4** and **5**, and >10 for compounds **6** and **8**, respectively. For the HSV-1 field strain, the EC_50_ values for the hit compounds were in the range 132.4–157.9 μM, with compounds **4**, **5**, **6** and **8** showing the best selectivity index (>7). For HSV-1 B2006, only compound **2** showed a significant difference in activity compared to the other hybrids with an EC_50_ value about half that of the other products (EC_50_ = 226.3 for compound **2** vs. EC_50_ between 105.9–122.1 for the other hybrids). 

Comparison of the results on the different viruses showed interesting information. For the pairs of compounds **1**–**2** and **7**–**8** (differing in one CH_2_ unit in the linker), activity was found for compounds **2** and **8**, suggesting that the length of the linker influences the antiviral effect of this group of compounds. However, no difference were observed against any of the three HSV-1 strains for the pairs IA-diterpene (compounds **3**–**8**, except for compound **7**). The hybrids IA-triterpene (compounds **9**–**16**) were inactive.

## 3. Experimental 

### 3.1. General Procedures 

Optical rotations were measured in CHCl_3_ at 20 °C on a Jasco DIP 370 polarimeter (Jasco Analytical Instruments, Easton, MD, USA). IR spectra were recorded on a Nicolet Nexus 470 FT-IR instrument (Thermo Electron Corporation, Whaltham, MA, USA). The NMR spectra were recorded on a Bruker Avance 400 spectrometer (Bruker, Rheinstetten, Germany) at 400 MHz for ^1^H and 100 MHz for ^13^C in CDCl_3_. Chemical shifts are given in ppm with TMS as the internal standard. High-resolution mass spectra were measured on a VG Micromass ZAB-2F at 70 eV (Varian Inc., Palo Alto, CA, USA). Merck silica gel (0.063–0.2) was used for column chromatography, pre-coated Si gel plates (Merck, Kieselgel 60 F_254_, 0.25 mm) were used for TLC analysis. TLC spots were visualized by spraying the chromatograms with *p*-anisaldehyde-ethanol-acetic acid-H_2_SO_4_ (2:170:20:10 *v*/*v*) and heating at 110 °C for 3 min. Reagents: *N*,*N*-Dicyclohexylcarbodiimide (DCC) and dimethylaminopyridine (DMAP) were from Merck (Schuchardt, Germany). Propargyl alcohol, 3-butyn-1-ol, 4-pentynoic acid and 5-hexynoic acid were from Aldrich (Steinheim, Germany). Copper (II) sulphate pentahydrate was from Aldrich (St. Louis, MO, USA) and sodium ascorbate was from Sigma (St. Louis, MO, USA).

### 3.2. Preparation of Derivatives

#### 3.2.1. Starting Compounds

Imbricatolic acid was isolated from the resin of *Araucaria araucana* [28]. Cyperenoic acid and acetyl aleuritolic acid were obtained from *Jatropha isabelli* [11]. Dehydroabietic acid was prepared from commercial rosin as previously described [33]. Carnosic acid was isolated from the aerial parts of *Rosmarinus officinalis* [35]. Ferruginol was isolated from the stem bark of *Prumnopitys andina* [36] and oleanolic acid from the aerial parts of *Fabiana imbricata* [37].

#### 3.2.2. Preparation of Terpene-Alkynyl Esters

Esterification of terpenes was performed using DCC/DMAP and an appropriate alcohol (propargyl alcohol or 3-butyn-1-ol) for cyperenoic acid, or acid (4-pentynoic or 5-hexynoic acid) for dehydroabietinol (obtained by reduction of dehydroabietic acid), carnosic acid, ferruginol, oleanolic acid and aleuritolic acid (obtained by deacetylation of acetyl aleuritolic acid) according to references [13,33,34]. In previous work we reported the formation of carnosic acid lactone in the esterification reaction leading to carnosic acid γ-lactone [35]. Briefly, cyperenoic acid or alkynyl acid (1 equiv.) was dissolved in dry CH_2_Cl_2_ at room temperature under constant stirring. Then, DCC (1 equiv.) was added, followed by a catalytic amount of DMAP and alkynyl alcohol or terpene alcohol (1 equiv.) dissolved in dry CH_2_Cl_2_. The reaction was stopped by adding H_2_O, extracted with CH_2_Cl_2_, dried over Na_2_SO_4_, concentrated and purified by column chromatography on silica gel (60–81% yield).

#### 3.2.3. Synthesis of Hybrid Derivatives **1**–**16**

The terpene-alkynyl esters (1 equiv.) and the IA-azide (1 equiv.) were dissolved in *t*-BuOH/H_2_O (1:1), followed by the addition of CuSO_4_·5H_2_O (2 mol %) and sodium ascorbate (10 mol %). The mixture was stirred at room temperature for 24 h. The reaction was stopped by adding H_2_O, extracted with CH_2_Cl_2_, dried over anhydrous Na_2_SO_4_, concentrated and purified by column chromatography on silica gel (53–83% yield). The compounds **10**, **12**, **14** and **16** were obtained by methylation using diazomethane in diethyl ether (Et_2_O) of the compounds **9**, **11**, **13** and **15**, respectively (88%–94% yield).

*Compound* (**1**). Colorless resin; [α]${}_{D}^{20}$ +21 (*c* 0.059, CHCl_3_); IR (film) ν_max_ 2949, 2864, 1720, 1707, 1660, 1456, 1255, 753 cm^−1^; ^1^H-NMR (400 MHz, CDCl_3_) δ 7.59 (s, 1H, H-1′), 5.24 (s, 2H, H-3′), 4.81 (s, 1H, H-17), 4.41 (s, 1H, H-17), 4.37–4.27 (m, 2H, H-15), 3.59 (s, 3H, OMe), 280–2.70 (m, 1H, H-3′′), 2.69–2.57 (m, 2H, H-3′′ and H-6′′), 2.39–2.36 (m, 1H, H-7), 2.20–2.10 (m, 2H, H-3 and H-6′′), 1.16 (s, 3H, H-18), 0.95 (s, 3H, H-13′′), 0.93 (d, *J* = 6.6 Hz, 3H, H-16), 0.80 (d, *J* = 6.5 Hz, 3H, H-14′′), 0.76 (s, 3H, H-12′′), 0.46 (s, 3H, H-20). ^13^C-NMR (CDCl_3_): δ 177.83, 170.67, 165.42, 148.33, 143.50, 123.73, 123.06, 106.38, 68.06, 57.27, 56.63, 56.42, 51.23, 48.67, 48.22, 44.38, 41.74, 40.45, 39.27, 38.85, 38.33, 37.18, 36.69, 35.97, 35.88, 31.30, 31.15, 28.92, 27.93, 27.02, 26.33, 25.80, 21.09, 20.05, 19.56, 19.36, 18.05, 12.65. HREIMS *m*/*z* 620.4486 [M + H]^+^ (calcd for C_38_H_58_N_3_O_4_, 620.4437). 

*Compound* (**2**). Colorless resin; [α]${}_{D}^{20}$ +18 (*c* 0.032, CHCl_3_); IR (film) ν_max_ 2927, 2867, 1722, 1704, 1663, 1462, 1258, 759 cm^−1^; ^1^H-NMR (400 MHz, CDCl_3_) δ 7.32 (s, 1H, H-1′), 4.82 (s, 1H, H-17), 4.42 (s, 1H, H-17), 4.41–4.28 (m, 4H, H-15 and H-4′), 3.60 (s, 3H, OMe), 3.10 (t, *J* = 6.7 Hz, 2H, H-3′), 2.82–2.70 (m, 1H, H-3′′), 2.68–2.54 (m, 2H, H-3′′ and H-6′′), 2.39–2.36 (m, 1H H-7), 2.17–2.13 (m, 2H, H-3 and H-6′′), 1.16 (s, 3H, H-18), 0.96 (s, 3H, H-13′′), 0.93 (d, *J* = 6.5 Hz, 3H H-16), 0.82 (d, *J* = 6.5 Hz, 3H, H-14′′), 0.78 (s, 3H, H-12′′), 0.47 (s, 3H, H-20). ^13^C-NMR (101 MHz, CDCl_3_) δ 177.86, 169.84, 165.44, 148.36, 144.51, 123.37, 121.27, 106.39,67.99, 62.70, 56.69, 56.45, 51.26, 48.57, 48.25, 44.41, 41.75, 40.48, 39.30, 38.88, 38.36, 37.38, 36.76, 35.96, 35.94, 31.28, 31.23, 28.94, 28.02, 27.08, 26.35, 25.85, 25.80, 21.11, 20.07, 19.59, 19.37, 18.11, 12.69. HREIMS *m*/*z* 656.4493 [M + Na]^+^ (calcd for C_39_H_59_N_3_NaO_4_, 656.4403).

*Compound* (**3**). Colorless resin; [α]${}_{D}^{20}$ +28 (*c* 0.050, CHCl_3_); IR (film) ν_max_ 2927, 2867, 1732, 1710, 1661, 1462, 1152, 756 cm^−1^; ^1^H-NMR (400 MHz, CDCl_3_) δ 7.28 (s, 1H, H-1′), 7.16 (d, *J* = 8.3 Hz, 1H, H-11′′), 6.98 (brd, *J* = 8.2 Hz, 1H, H-12′′), 6.88 (s, 1H, H-14′′), 4.82 (s, 1H, H-17), 4.44 (s, 1H, H-17), 4.32–4.22 (m, 2H, H-15), 3.96 (d, *J* = 10.9 Hz, 1H, H-18′′), 3.73 (d, *J* = 10.9 Hz, 1H, H-18′′), 3.60 (s, 3H, OMe), 3.01 (t, *J* = 7.3 Hz, 2H, H-4′), 2.93–2.62 (m, 3H, H-7′′ and H-15′′), 2.71 (t, *J* = 7.4 Hz, 2H, H-3′), 2.40–2.37 (m, 1H, H-7), 2.32–2.23 (m, 1H, H-1β′′), 2.18–2.15 (m, 1H, H-3), 1.22 (d, *J* = 6.9 Hz, 6H, H-16′′ and H-17′′), 1.20 (s, 3H, H-20′′), 1.17 (s, 3H, H-18), 0.93 (d, *J* = 6.5 Hz, 3H, H-16), 0.92 (s, 3H, H-19′′), 0.48 (s, 3H, H-20). ^13^C-NMR (101 MHz, CDCl_3_) δ 177.83, 172.98, 148.33, 147.14, 146.34, 145.67, 134.71, 126.95, 124.36, 123.98, 121.00, 106.39, 72.76, 56.64, 56.43, 51.21, 48.47, 44.50, 44.39, 40.45, 39.27, 38.85, 38.34, 37.49, 37.25, 36.88, 35.91, 35.64, 33.80, 33.51, 31.13, 30.32, 28.91, 26.34, 25.44, 24.06 (2C), 21.14, 21.08, 20.05, 19.56, 19.10, 18.60, 17.50, 12.66. HREIMS *m*/*z* 714.5302 [M + H]^+^ (calcd for C_45_H_68_N_3_O_4_, 714.5210).

*Compound* (**4**). Colorless resin; [α]${}_{D}^{20}$ +26 (*c* 0.191, CHCl_3_); IR (film) ν_max_ 2930, 2867, 1722, 1718, 1678, 1462, 1155, 753 cm^−1^; ^1^H-NMR (400 MHz, CDCl_3_) δ 7.17 (d, *J* = 8.1 Hz, 1H, H-11′′), 7.14 (s, 1H, H-1′), 6.97 (brd, *J* = 8.1 Hz, 1H, H-12′′), 6.86 (s, 1H, H-14′′), 4.80 (s, 1H, H-17), 4.42 (s, 1H, H-17), 4.37–4.17 (m, 2H, H-15), 3.97 (d, *J* = 11.0 Hz, 1H, H-18′′), 3.67 (d, *J* = 11.0 Hz, 1H, H-18′′), 3.58 (s, 3H, OMe), 2.94–2.58 (m, 5H, H-7′′, H-15′′ and H-5′), 2.42–2.21 (m, 4H, H-7, H-1β′′ and H-3′), 2.19–2.15 (m, 1H, H-3), 1.19 (d, *J* = 6.5 Hz, 6H, H-16′′ and H-17′′), 1.18 (s, 3H, H-20′′), 1.15 (s, 3H, H-18), 0.92 (d, *J* = 6.5 Hz, 3H, H-16), 0.91 (s, 3H, H-19′′), 0.46 (s, 3H, H-20). ^13^C-NMR (101 MHz, CDCl_3_) δ 177.73, 173.41, 148.26, 147.14, 146.97, 145.61, 134.71, 126.90, 124.28, 123.91, 120.68, 106.32, 72.37, 56.58, 56.36, 51.13, 48.38, 44.31, 40.38, 39.20, 38.79, 38.34, 38.27, 37.44, 37.21, 36.83, 35.85, 35.59, 33.59, 33.44, 31.09, 30.25, 28.85, 26.28, 25.39, 24.97, 24.68, 23.99 (2C), 21.03, 19.99, 19.52, 19.01, 18.55, 17.52, 12.59. HREIMS *m*/*z* 728.5318 [M + H]^+^ (calcd for C_46_H_70_N_3_O_4_, 728.5366). 

*Compound* (**5**). Colorless resin; [α]${}_{D}^{20}$ +27 (*c* 0.053, CHCl_3_); IR (film) ν_max_ 2927, 2870, 1754, 1718, 1641, 1462, 1136, 756 cm^−1^; ^1^H-NMR (400 MHz, CDCl_3_) δ 7.37 (s, 1H, H-1′), 6.91 (s, 1H, H-14′′), 6.78 (s, 1H, H-11′′), 4.82 (s, 1H, H-17), 4.43 (s, 1H, H-17), 4.34–4.26 (m, 2H, H-15), 3.61 (s, 3H, OMe), 3.15 (t, *J =* 7.2 Hz, 2H, H-4′), 3.00 (t, *J* = 7.2 Hz, 2H, H-3′), 2.93–2.87 (m, 1H, H-15′′), 2.84–2.73 (m, 2H, H-7′′), 2.44–2.35 (m, 1H, H-7), 2.21–2.08 (m, 2H, H-3 and H-1β′′), 1.17 (s, 3H, H-18), 1.16 (s, 3H, H-20′′), 1.12 (d, *J =* 6.9 Hz, 3H, H-16′′), 1.10 (d, *J* = 6.9 Hz, 3H, H-17′′), 0.94 (d, *J* = 6.6 Hz, 3H, H-16), 0.93 (s, 3H, H-18′′), 0.91 (s, 3H, H-19′′), 0.48 (s, 3H, H-20). ^13^C-NMR (101 MHz, CDCl_3_) δ 177.90, 171.96, 148.88, 148.36, 146.21, 146.09, 136.62, 133.24, 126.97, 121.40, 117.95, 106.43, 56.65, 56.44, 51.28, 50.12, 48.57, 44.41, 41.74, 40.48, 39.29, 38.88, 38.36, 37.68, 37.31, 35.92, 33.89, 33.52, 33.39, 31.22, 30.08, 28.95, 27.14, 26.37, 24.93, 23.15, 23.00, 21.70, 21.15, 21.10, 20.07, 19.61, 19.32, 19.13, 12.68. HREIMS *m*/*z* 714.5292 [M + H]^+^ (calcd for C_45_H_68_N_3_O_4_, 714.5210).

*Compound* (**6**). Colorless resin; [α]${}_{D}^{20}$ +33 (*c* 0.085, CHCl_3_); IR (film) ν_max_ 2949, 2864, 1754, 1719, 1678, 1459, 1124, 750 cm^−1^; ^1^H-NMR (400 MHz, CDCl_3_) δ 7.31 (s, 1H, H-1′), 6.93 (s, 1H, H-14′′), 6.80 (s, 1H, H-11′′), 4.82 (s, 1H, H-17), 4.44 (s, 1H, H-17), 4.36–4.25 (m, 2H, H-15), 3.60 (s, 1H, OMe), 2.93–2.75 (m, 3H, H-15′′, H-7′′), 2.63 (t, *J* = 7.4 Hz, 2H, H-5′), 2.38 (m, 1H, H-7), 2.17–2.10 (m, 2H, H-3 and H-1β′′), 2.14 (t, *J* = 7.4 Hz, 2H, H-3′), 1.17 (s, 3H, H-18), 1.16 (s, 3H, H-20′′), 1.16 (d, *J =* 6.6 Hz, 3H, H-16′′), 1.15 (d, *J =* 6.6 Hz, 3H, H-17′′), 0.94 (d, *J* = 6.6 Hz, 3H, H-16), 0.93 (s, 3H, H-18′′), 0.90 (s, 3H, H-19′′), 0.47 (s, 1H, H-20). ^13^C-NMR (101 MHz, CDCl_3_) δ 177.86, 172.29, 148.86, 148.37, 147.08, 146.15, 136.62, 133.12, 126.91, 120.82, 118.00, 106.39, 56.66, 56.44, 51.24, 50.13, 48.54, 44.40, 41.73, 40.47, 39.28, 38.87, 38.35, 37.67, 37.31, 35.93, 33.70, 33.51, 33.37, 31.19, 30.06, 28.93, 27.18, 26.35, 25.09, 24.89, 24.82, 23.20, 23.05, 21.68, 21.12, 20.07, 19.60, 19.30, 19.13, 12.67. HREIMS *m*/*z* 728.5332 [M + H]^+^ (calcd for C_46_H_70_N_3_O_4_, 728.5366). 

*Compound* (**7**). Colorless resin; [α]${}_{D}^{20}$ +57 (*c* 0.122, CHCl_3_); IR (film) ν_max_ 2955, 2867, 1798, 1760, 1722, 1648, 1440, 1123, 756 cm^−1^; ^1^H-NMR (400 MHz, CDCl_3_) δ 7.49 (s, 1H, H-1′), 6.68 (s, 1H, H-14′′), 4.77 (s, 1H, H-17), 4.41 (s, 1H, H-17), 4.35–4.22 (m, 2H, H-15), 3.56 (s, 3H, OMe), 3.15 (t, *J* = 6.6 Hz, 2H H-4′), 2.97 (t, *J* = 7.5 Hz, 2H, H-3′), 2.95–2.89 (m, 1H, H-15′′), 2.59 (m, 2H, H-7′′), 2.36–2.32 (m, 1H, H-7), 1.15 (d, *J* = 7.3 Hz, 3H, H-16′′), 1.14 (s, 3H, H-18), 1.11 (s, 3H, H-18′′), 1.08 (d, *J* = 6.9 Hz, 3H, H-17′′), 1.04 (s, 3H, H-19′′), 0.90 (d, *J* = 6.5 Hz, 3H, H-16), 0.44 (s, 3H, H-20). ^13^C-NMR (101 MHz, CDCl_3_) δ 177.71, 177.49, 170.20, 148.17, 144.53, 140.83, 137.25, 130.35, 129.38, 121.53, 119.97, 106.35, 56.54, 56.43, 56.34, 51.09, 49.57, 48.48, 44.29, 41.58, 40.35, 39.16, 38.77, 38.65, 38.26, 37.10, 35.83, 33.60, 32.85, 32.76, 31.83, 31.06, 28.83, 27.69, 26.25, 23.92, 23.46, 22.54, 22.17, 21.17, 20.98, 19.97, 19.45, 18.18, 12.56. HREIMS *m*/*z* 742.4739 [M + H]^+^ (calcd for C_45_H_64_N_3_O_6_, 742.4795).

*Compound* (**8**). Colorless resin; [α]${}_{D}^{20}$ +56 (*c* 0.122, CHCl_3_); IR (film) ν_max_ 2949, 2864, 1798, 1763, 1720, 1650, 1440, 1123, 756 cm^−1^; ^1^H-NMR (400 MHz, CDCl_3_) δ 7.39 (s, 1H, H-1′), 6.69 (s, 1H, H-14′′), 4.78 (s, 1H, H-17), 4.40 (s, 1H, H-17), 4.40–4.25 (m, 2H, H-15), 3.56 (s, 3H, OMe), 2.97 (m, 1H, H-15′′), 2.84 (t, *J* = 7.3 Hz, 2H, H-5′), 2.62 (t, *J* = 7.3 Hz, 2H, H-3′), 2.61–2.58 (m, 2H, H-7′′), 2.39–2.29 (m, 1H, H-7), 1.18 (d, *J* = 6.9 Hz, 3H H-16′′), 1.13 (s, 3H, H-18), 1.12 (d, *J* = 6.9 Hz, 3H H-17′′), 1.11 (s, 3H, H-18′′), 1.04 (s, 3H, H-19′′), 0.91 (d, *J* = 6.5 Hz, 3H, H-16), 0.44 (s, 1H, H-20). ^13^C-NMR (101 MHz, CDCl_3_) δ 177.69, 177.63, 170.65, 148.18, 144.60, 140.87, 137.15, 130.36, 129.48, 121.23, 119.91, 106.33, 56.55, 56.42, 56.33, 51.09, 49.59, 48.40, 44.28, 41.55, 40.35, 39.16, 38.76, 38.63, 38.25, 37.13, 35.83, 32.88, 32.84, 32.77, 31.82, 31.05, 28.82, 27.68, 26.25, 24.59, 24.54, 23.93, 23.52, 22.60, 22.17, 20.98, 19.96, 19.48, 18.17, 12.57. HREIMS *m*/*z* 756.4894 [M + H]^+^ (calcd for C_46_H_66_N_3_O_6_, 756.4952).

*Compound* (**9**). White solid; [α]${}_{D}^{20}$ +30 (*c* 0.052, CHCl_3_); IR (film) ν_max_ 3407, 2949, 2870, 1720, 1691, 1462, 1152, 756 cm^−1^; ^1^H-NMR (400 MHz, CDCl_3_) δ 7.30 (s, 1H, H-1′), 5.25 (brs, 1H, H-12′′), 4.82 (s, 1H, H-17), 4.47 (t, *J* = 7.9 Hz, 1H, H-3α′′), 4.43 (s, 1H, H-17), 4.38–4.25 (m, 2H, H-15), 3.60 (s, 3H, OMe), 3.03 (t, *J* = 7.3 Hz, 2H, H-4′), 2.80 (dd, *J* = 13.6, 3.7 Hz, 1H, H-18′′), 2.71 (t, *J* = 7.0 Hz, 2H, H-3′), 2.39–2.36 (m, 1H, H-7), 2.17–2.14 (m, 1H, H-3), 1.17 (s, 3H, H-18), 1.11 (s, 3H), 0.93 (d, *J* = 6.6 Hz, 3H, H-16), 0.91 (s, 6H), 0.89 (s, 3H), 0.80 (s, 3H), 0.78 (s, 3H), 0.72 (s, 3H), 0.47 (s, 3H, H-20). ^13^C-NMR (101 MHz, CDCl_3_) δ 183.93, 177.87, 172.72, 148.36, 146.51, 143.76, 122.58, 121.05, 106.42, 81.21, 56.67, 56.45, 55.41, 51.28, 48.53, 47.65, 46.62, 45.96, 44.41, 41.66, 41.02, 40.48, 39.37, 39.30, 38.89, 38.36, 38.15, 37.82, 37.30, 37.07, 35.96, 34.23, 33.89, 33.18, 32.63, 32.54, 31.16, 30.78, 28.95, 28.11, 27.77, 26.36, 26.02, 23.69, 23.65, 23.49, 22.98, 21.29, 21.09, 20.08, 19.60, 18.26, 17.24, 16.80, 15.47, 12.69. HREIMS *m*/*z* 898.6106 [M + H]^+^ (calcd for C_56_H_88_N_3_O_6_, 898.6673).

*Compound* (**10**). White solid; [α]${}_{D}^{20}$ +26 (*c* 0.064, CHCl_3_); IR (film) ν_max_ 2938, 2875, 1724, 1458, 1157, 752 cm^−1^; ^1^H-NMR (400 MHz, CDCl_3_) δ 7.29 (s, 1H, H-1′), 5.26 (brs, 1H, H-12′′), 4.81 (s, 1H, H-17), 4.47 (t, *J* = 7.9 Hz, 1H, H-3α′′), 4.42 (s, 1H, H-17), 4.38–4.24 (m, 2H, H-15), 3.60 (s, 3H, OMe), 3.59 (s, 3H, OMe), 3.02 (t, *J* = 7.3 Hz, 2H, H-4′), 2.84 (dd, *J* = 13.7, 3.6 Hz, 1H, H-18′′), 2.70 (t, *J* = 7.0 Hz, 2H, H-3′), 2.39–2.36 (m, 1H, H-7), 2.17–2.13 (m, 1H, H-3), 1.16 (s, 3H, H-18), 1.10 (s, 3H), 0.92 (d, *J* = 6.6 Hz, 3H, H-16), 0.90 (s, 6H), 0.88 (s, 3H), 0.80 (s, 3H), 0.77 (s, 3H), 0.70 (s, 3H), 0.46 (s, 3H, H-20). ^13^C-NMR (101 MHz, CDCl_3_) δ 178.37, 177.83, 172.67, 148.35, 146.50, 143.91, 122.34, 120.98, 106.40, 81.20, 56.65, 56.43, 55.41, 51.62, 51.25, 48.49, 47.64, 46.80, 45.94, 44.39, 41.72, 41.38, 40.46, 39.37, 39.28, 38.87, 38.35, 38.18, 37.80, 37.28, 37.01, 35.94, 34.24, 33.94, 33.20, 32.68, 32.46, 31.13, 30.78, 28.94, 28.09, 27.77, 26.35, 26.00, 23.74, 23.64, 23.49, 23.15, 21.31, 21.08, 20.07, 19.59, 18.29, 16.92, 16.80, 15.44, 12.67. HREIMS *m*/*z* 934.6866 [M + Na]^+^ (calcd for C_57_H_89_N_3_NaO_6_, 934.6649).

*Compound* (**11**). White solid; [α]${}_{D}^{20}$ +40 (*c* 0.123, CHCl_3_); IR (film) ν_max_ 3406, 2943, 2871, 1719, 1688, 1458, 1150, 752 cm^−1^; ^1^H-NMR (400 MHz, CDCl_3_) δ 7.27 (s, 1H, H-1′), 5.24 (brs, 1H, H-12′′), 4.80 (s, 1H, H-17), 4.48 (t, *J* = 7.9 Hz, 1H, H-3α′′), 4.41 (s, 1H, H-17), 4.36–4.24 (m, 2H, H-15), 3.58 (s, 3H, OMe), 2.80 (dd, *J* = 13.7, 4.0 Hz, 1H, H-18′′), 2.73 (t, *J* = 7.5 Hz, 2H, H-5′), 2.39–2.37 (s, 1H, H-7), 2.34 (t, *J* = 7.3 Hz, 2H, H-3′), 2.16–2.12 (m, 1H, H-3), 1.15 (s, 3H, H-18), 1.10 (s, 3H), 0.92 (d, *J* = 6.6 Hz, 3H, H-16), 0.90 (s, 6H), 0.87 (s, 3H), 0.83 (s, 3H), 0.82 (s, 3H), 0.72 (s, 3H), 0.45 (s, 3H, H-20). ^13^C-NMR (101 MHz, CDCl_3_) δ 184.00, 177.85, 173.24, 148.32, 147.18, 143.74, 122.52, 120.78, 106.38, 80.93, 56.60, 56.39, 55.34, 51.24, 48.50, 47.61, 46.57, 45.92, 44.36, 41.62, 40.96, 40.43, 39.33, 39.24, 38.84, 38.31, 38.10, 37.79, 37.26, 37.04, 35.89, 34.10, 33.86, 33.15, 32.58, 32.50, 31.13, 30.75, 28.91, 28.17, 27.73, 26.32, 25.99, 25.03, 24.92, 23.66, 23.64, 23.45, 22.93, 21.07, 20.03, 19.57, 18.23, 17.20, 16.83, 15.45, 12.65. HREIMS *m*/*z* 934.6572 [M + Na]^+^ (calcd for C_57_H_89_N_3_NaO_6_, 934.6649).

*Compound* (**12**). White solid; [α]${}_{D}^{20}$ +19 (*c* 0.012, CHCl_3_); IR (film) ν_max_ 2947, 2862, 1724, 1454, 1153, 752 cm^−1^; ^1^H-NMR (400 MHz, CDCl_3_) δ 7.27 (s, 1H, H-1′), 5.27 (brs, 1H, H-12′′), 4.82 (s, 1H, H-17), 4.49 (t, *J* = 7.9 Hz, 1H, H-3α′′), 4.43 (s, 1H, H-17), 4.36–4.25 (m, 2H, H-15), 3.61 (s, 3H, OMe), 3.60 (s, 3H, OMe), 2.85 (dd, *J* = 13.6, 3.8 Hz, 1H, H-18′′), 2.75 (t, *J* = 7.5 Hz, 2H, H-5′), 2.40–2.37 (s, 1H, H-7), 2.36 (t, *J* = 7.2 Hz, 2H, H-3′), 2.17–2.14 (m, 1H, H-3), 1.17 (s, 3H, H-18), 1.12 (s, 3H), 0.94 (d, *J* = 6.6 Hz, 3H, H-16), 0.91 (s, 6H), 0.89 (s, 3H), 0.84 (s, 6H), 0.71 (s, 3H), 0.47 (s, 3H, H-20). ^13^C-NMR (101 MHz, CDCl_3_) δ 178.45, 177.91, 173.27, 148.40, 147.27, 143.92, 122.37, 120.76, 106.41, 80.99, 56.66, 56.44, 55.40, 51.67, 51.29, 48.53, 47.66, 46.83, 45.95, 44.41, 41.74, 41.39, 40.49, 39.39, 39.30, 38.89, 38.36, 38.19, 37.85, 37.32, 37.04, 35.95, 34.17, 33.97, 33.23, 32.69, 32.49, 31.19, 30.81, 28.96, 28.22, 27.79, 26.37, 26.02, 25.13, 24.98, 23.76, 23.70, 23.52, 23.17, 21.13, 20.08, 19.62, 18.32, 16.94, 16.90, 15.48, 12.69. HREIMS *m*/*z* 926.7061 [M + H]^+^ (calcd for C_58_H_92_N_3_O_6_, 926.6986).

*Compound* (**13**). White solid; [α]${}_{D}^{20}$ +24 (*c* 0.062, CHCl_3_); IR (film) ν_max_ 3433, 2934, 2857, 1733, 1679, 1472, 1153, 752 cm^−1^; ^1^H-NMR (400 MHz, CDCl_3_) δ 7.30 (s, 1H, H-1′), 5.49 (dd, *J* = 7.7, 3.1 Hz, 1H, H-15′′), 4.81 (s, 1H, H-17), 4.46–4.42 (m, 1H, H-3′′), 4.42 (s, 1H, H-17), 4.34–4.22 (m, 2H, H-15), 3.58 (s, 3H, OMe), 3.01 (t, *J* = 7.3 Hz, 2H, H-4′), 2.69 (t, *J* = 7.0 Hz, 2H, H-3′), 2.39–2.32 (m, 2H, H-7 and H-16′′, 2.27–2.23 (m, 1H, H-18′′), 2.16–2.13 (m, 1H, H-3), 1.16 (s, 3H, H-18), 0.92 (d, *J* = 6.6 Hz, 3H, H-16), 0.91 (s, 6H), 0.90 (s, 3H), 0.89 (s, 6H), 0.83 (s, 3H), 0.76 (s, 3H), 0.46 (s, 3H, H-20). ^13^C-NMR (101 MHz, CDCl_3_) δ 184.45, 177.83, 172.65, 160.53, 148.31, 146.45, 121.03, 116.98, 106.40, 81.11, 56.61, 56.40, 55.64, 51.56, 51.25, 49.12, 48.49, 44.36, 41.40, 40.78, 40.44, 39.25, 39.08, 38.85, 38.33, 37.97, 37.77, 37.41, 37.36, 37.26, 35.90, 35.40, 34.18, 33.73, 33.37, 31.94, 31.37, 31.09, 30.76, 29.38, 28.92, 28.74, 28.01, 26.32, 26.27, 23.54, 22.56, 21.25, 21.04, 20.04, 19.56, 18.77, 17.39, 16.70, 15.70, 12.65. HREIMS *m*/*z* 898.6540 [M + H]^+^ (calcd for C_56_H_88_N_3_O_6_, 898.6673).

*Compound* (**14**). Colorless resin; [α]${}_{D}^{20}$ +22 (*c* 0.056, CHCl_3_); IR (film) ν_max_ 2943, 2871, 1724, 1454, 1166, 752 cm^−1^; ^1^H-NMR (400 MHz, CDCl_3_) δ 7.29 (s, 1H, H-1′), 5.48 (dd, *J* = 7.9, 3.3 Hz, 1H, H-15′′), 4.81 (s, 1H, H-17), 4.46–4.43 (m, 1H, H-3′′), 4.43 (s, 1H, H-17), 4.31–4.24 (m, 2H, H-15), 3.60 (s, 3H, OMe), 3.55 (s, 3H, OMe), 3.02 (t, *J* = 7.3 Hz, 2H, H-4′), 2.70 (t, *J* = 7.7 Hz, 2H, H-3′), 2.34–2.34 (m, 2H, H-7 and H-16′′, 2.32–2.29 (m, 1H, H-18′′), 2.17–2.14 (m, 1H, H-3), 1.17 (s, 3H, H-18), 0.93 (d, *J* = 6.6 Hz, 3H, H-16), 0.92 (s, 3H), 0.91 (s, 6H), 0.90 (s, 6H), 0.82 (s, 3H), 0.76 (s, 3H), 0.47 (s, 3H, H-20). ^13^C-NMR (101 MHz, CDCl_3_) δ 178.57, 177.85, 172.67, 160.61, 148.37, 146.52, 120.99, 116.71, 106.42, 81.26, 56.69, 56.47, 55.76, 51.80, 51.47, 51.25, 49.15, 48.51, 44.42, 41.97, 41.07, 40.49, 39.31, 39.08, 38.90, 38.38, 37.97, 37.82, 37.52, 37.50, 37.29, 35.96, 35.60, 34.25, 33.87, 33.52, 32.18, 31.78, 31.15, 31.10, 29.39, 28.95, 28.83, 28.03, 26.37, 26.36, 23.59, 22.50, 21.32, 21.09, 20.09, 19.60, 18.70, 17.43, 16.69, 15.62, 12.69. HREIMS *m*/*z* 912.6757 [M + H]^+^ (calcd for C_57_H_90_N_3_O_6_, 912.6830).

*Compound* (**15**). White solid; [α]${}_{D}^{20}$ +26 (*c* 0.044, CHCl_3_); IR (film) ν_max_ 3433, 2938, 2862, 1728, 1679, 1459, 1153, 757 cm^−1^; ^1^H-NMR (400 MHz, CDCl_3_) δ 7.24 (s, 1H, H-1′), 5.50 (dd, *J* = 7.7, 3.1 Hz, 1H, H-15′′), 4.80 (s, 1H, H-17), 4.44–4.41 (m, 1H, H-3′′), 4.41 (s, 1H, H-17), 4.33–4.23 (m, 2H, H-15), 3.58 (s, 3H, OMe), 2.70 (t, *J* = 7.5 Hz, 2H, H-5′), 2.40–2.22 (m, 5H, H-7, H-16′′, H-5′ and H-18′′), 2.16–2.12 (m, 1H, H-3), 1.15 (s, 3H, H-18), 0.92 (s, 6H), 0.91 (d, *J* = 6.6 Hz, 3H, H-16), 0.90 (s, 3H), 0.89 (s, 6H), 0.84 (s, 3H), 0.81 (s, 3H), 0.46 (s, 3H, H-20). ^13^C-NMR (101 MHz, CDCl_3_) δ 184.28, 177.87, 173.47, 160.63, 148.39, 147.80, 120.54, 116.97, 106.40, 80.82, 56.66, 56.46, 55.70, 51.57, 51.26, 49.18, 48.51, 44.41, 41.49, 40.87, 40.48, 39.30, 39.14, 38.89, 38.37, 38.03, 37.81, 37.48, 37.41, 37.32, 35.94, 35.46, 34.50, 33.79, 33.42, 31.98, 31.44, 31.20, 30.82, 29.41, 28.97, 28.95, 28.77, 28.12, 26.37, 26.29, 25.45, 24.71, 22.58, 21.13, 20.09, 19.61, 18.82, 17.43, 16.77, 15.74, 12.69. HREIMS *m*/*z* 934.6755 [M + Na]^+^ (calcd for C_57_H_89_N_3_NaO_6_, 934.6649).

*Compound* (**16**). Colorless resin; [α]${}_{D}^{20}$ +40 (*c* 0.030, CHCl_3_); IR (film) ν_max_ 2938, 2862, 1724, 1454, 1157, 752 cm^−1^; ^1^H-NMR (400 MHz, CDCl_3_) δ 7.24 (s, 1H, H-1′), 5.49 (dd, *J* = 7.9, 3.3 Hz, 1H, H-15′′), 4.82 (s, 1H, H-17), 4.47–4.43 (m, 1H, H-3′′), 4.44 (s, 1H, H-17), 4.33–4.26 (m, 2H, H-15), 3.60 (s, 3H, OMe), 3.56 (s, 3H, OMe), 2.72 (t, *J* = 6.8 Hz, 1H, H-5′), 2.41–2.31 (m, 5H, H-7, H-16′′, H-5′ and H-18′′), 2.18–2.15 (m, 1H, H-3), 1.17 (s, 3H, H-18), 0.94 (d, *J* = 6.6 Hz, 3H, H-16), 0.93 (s, 3H), 0.92 (s, 9H), 0.91 (s, 3H), 0.84 (s, 3H), 0.82 (s, 3H), 0.48 (s, 3H, H-20). ^13^C-NMR (101 MHz, CDCl_3_) δ 178.62, 177.90, 173.48, 160.65, 148.43, 147.85, 124.43, 120.52, 116.72, 106.41, 80.93, 56.70, 56.49, 55.77, 51.83, 51.49, 51.28, 49.16, 48.52, 44.44, 41.99, 41.10, 40.51, 39.33, 39.11, 38.92, 38.40, 38.01, 37.84, 37.52, 37.35, 36.77, 35.97, 35.62, 34.58, 33.89, 33.55, 32.20, 31.81, 31.22, 31.13, 29.41, 29.01, 28.97, 28.85, 28.14, 26.40, 26.38, 25.49, 24.75, 22.52, 21.15, 20.11, 19.64, 18.73, 17.45, 16.77, 15.65, 12.71. HREIMS *m*/*z* 926.7031 [M + H]^+^ (calcd for C_58_H_92_N_3_O_6_, 926.6986). 

### 3.3. Treatment Solutions

The synthetic compounds and acyclovir (ACV, Sigma) were dissolved in dimethylsulfoxide (DMSO) and diluted with Eagle’s minimal essential medium supplemented with 1.5% inactivated fetal bovine serum (FBS) and 50 μg/mL gentamicin (MEM 1.5%). The maximum concentration of DMSO used (1%) exhibited no toxicity under in vitro conditions. 

### 3.4. Cells and Viruses

Vero and Hep-2 cells were grown in MEM supplemented with 10% inactivated FBS and 50 µg/mL gentamicin and maintained in MEM 1.5%. Raw 264.7 cells were grown in Dulbecco’s Modified Eagle’s Medium (DMEM) supplemented with 10% FBS and 50 µg/mL gentamicin and maintained in DMEM 1.5%. C6 cells were grown in DMEM-F12 supplemented with 2.5% FBS50 µg/mL gentamicin and 15% horse serum (HS) and maintained in DMEM-F12 supplemented with 1.5% FBS. HSV-1 KOS, thymidine kinase-deficient (TK−) B2006 and Field strains of HSV-1 were used and propagated at low multiplicity of infection (m.o.i.) on Vero cells.

### 3.5. Cytotoxicity Assay

Cell viability was determined using the tetrazolium salt MTT (3-(4,5-dimethylthiazol-2-yl)-2,5-diphenyltetrazolium bromide, Sigma) according to the manufacturer’s instructions. Hep-2, C6, Raw 264.7 and Vero cells were seeded at a concentration of 1 × 10^4^ cells/well in 96-well plates and grown at 37 °C for 24 h. The culture medium was replaced by medium containing the compounds in triplicate, and cells were incubated for 48 h. The absorbance was measured on a MPR-A 4i microplate reader (Eurogenetics, Tipperary, Ireland) using a test wavelength of 570 nm and a reference wavelength of 630 nm. Results were expressed as a percentage of absorbance of treated cell cultures with respect to untreated ones. The cytotoxic concentration 50 (CC_50_) was calculated as the concentration of compounds required to reduce cell viability by 50% relative to untreated cells. 

### 3.6. Cytopathic Effect Assay 

Cells grown in 96-well plates were infected or not with HSV-1 (KOS, Field and B2006 strains) at a m.o.i. of 0.1 PFU/cell. After 1-h adsorption at 37 °C, the inoculum was removed and medium containing or not the compounds was added, in triplicate. The plates were incubated at 37 °C until 48 h post-infection (p.i.), when 100% of cells were death in virus control, due to the cytophatic effect. Then, cells were fixed with 10% formaldehyde for 15 min at room temperature, washed once with distilled water and stained with 0.05% crystal violet in 10% ethanol over 30 min. Afterward, cells were washed once and eluted with a solution of 50% ethanol and 0.1% acetic acid in water. The absorbance of each well was measured on an Eurogenetics MPR-A 4i microplate reader using a test wave-length of 590 nm. Results were analyzed as the percentage of absorbance of treated and infected cells compared with control (untreated/uninfected) cells. We considered the untreated/uninfected control cells as 100% of cell survival. The compound concentration that inhibit 50% viral cytopathic effect (EC_50_) was calculated. 

### 3.7. Statistical Analysis

The data were expressed as the mean ± standard deviation (SD) of three independent experiments.

## 4. Conclusions

To expand the structural diversity of naturally occurring terpenes in the search for new compounds of biological interest, sixteen hybrid compounds were synthetized. Using click reactions the diterpene imbricatolic acid (IA) containing an azide group and different terpenes (cyperenoic acid, dehydroabietinol, carnosic acid γ-lactone, ferruginol, oleanolic acid and aleuritolic acid) containing alkyne groups were fused. The cytotoxic activity of the hybrids was assessed against Vero and selected tumour cell lines. The compounds **1**, **2**, **3** and **7** were the most cytotoxic against the cell lines tested. The antiviral activity of the compounds was evaluated against the KOS, Field and B2006 strain of HSV-1. For the pairs of hybrid compounds **3**–**8** (except for compound **7**), a moderate activity was observed against the three HSV-1 strains with interesting selectivity index for some compounds.

Little information exists in bibliography on the possible activity of fused terpenes so this technique can be applied to a wide range of molecules (terpenes or not) to obtain new hybrid molecules with enhanced pharmacological action.

## Figures and Tables

**Table 1:** Cytotoxic activity of compounds **1**–**4** and **7**–**8** against Vero and selected tumor cell lines *^a^*.

Compound	CC_50_ (μM)			
	Vero	HEP-2	C6	Raw 264.7
**1**	623.9 ± 5	229.7 ± 1.8	>1000	>1000
**2**	216.2 ± 2	48.1 ± 1.1	354.9 ± 2.5	300 ± 1.9
**3**	667.5 ± 4.2	411.8 ± 2.1	>1000	>1000
**4**	>1000	>1000	>1000	>1000
**7**	680 ± 4.8	700 ± 5.8	552.5 ± 4	552.5 ± 3.9
**8**	>1000	>1000	>1000	>1000

*^a^* For the cell lines used, see the text.

**Table 2:** Antiviral activity of hit compounds against wild-type and ACV-resistant HSV-1 strains. EC_50_s were calculated by nonlinear regression.

Compound	CC_50_ *^a^* (μM)	EC_50_ (μM)			SI		
		KOS	Field	B2006	KOS	Field	B2006
**2**	216.2 ± 2	118.4 ± 2.3	157.9 ± 1.9	226.3 ± 2.1	1.8	1.3	0.9
**3**	667.5 ± 4.2	107.9 ± 1.2	140.2 ± 1.2	122.1 ± 1.2	6.2	4.76	5.5
**4**	>1000	109 ± 1.0	137.5 ± 1.9	110 ± 1.0	>9.2	>7.2	>9
**5**	>1000	105.1 ± 1.1	140.3 ± 2.1	112.1 ± 1.1	>9.5	>7.1	>8.9
**6**	>1000	96.2 ± 1.2	137.8 ± 1.4	120 ± 1.5	>10.4	>7.3	>8.3
**8**	>1000	99.3 ± 1.5	132.4 ± 1.1	105.9 ± 1.9	>10	>7.6	>9.4
ACV *^b^*	>1000	8.2 ± 1.0	73 ± 1.0	50 ± 2.0	>121.9	>13.7	>20

*^a^* For Vero cells. *^b^* ACV**:** Acyclovir, reference compound.
